# Impact on symptoms and survival of bone metastases in patients with small‐intestinal neuroendocrine tumours

**DOI:** 10.1111/jne.70073

**Published:** 2025-08-08

**Authors:** Maria Wedin, Eva Tiensuu Janson, Göran Wallin, Anders Sundin, Kosmas Daskalakis

**Affiliations:** ^1^ Department of Surgery Faculty of Medicine and Health, Örebro University Örebro Sweden; ^2^ Department of Medical Sciences Endocrine Oncology unit, Uppsala University Uppsala Sweden; ^3^ Department of Surgical Sciences Radiology and Molecular Imaging, Uppsala University Uppsala Sweden; ^4^ Second Department of Surgery ‘Korgialenio‐Benakio’, Red Cross General Hospital Athens Greece

**Keywords:** bone metastases, overall survival, small intestinal neuroendocrine tumours

## Abstract

We aimed to assess the symptoms and impact on overall survival (OS) from bone metastases (BM) diagnosed on Gallium‐68‐labelled DOTA tyrosine octreotide positron emission tomography with computed tomography (^68^Ga‐DOTATOC‐PET/CT) in patients with well‐differentiated small intestinal neuroendocrine tumours (Si‐NETs). Patients with well‐differentiated Si‐NETs, who underwent ^68^Ga‐DOTATOC‐PET/CT between 2010 and 2023 at two tertiary referral centres in Sweden, were included. Their number of BM, ≤5 BM versus >5 BM, symptoms and need for analgesics were recorded. To further assess the impact of BM on OS, we used a control group of age‐ and sex‐matched Si‐NET patients with liver metastases (Stage IV disease) but without BM. The prevalence of BM in Si‐NET patients was 23% (175/753); among these, complete clinical data were available in 138 patients. Synchronous BM were found in 33% (46/138). Sixty‐one patients (44%) showed >5 BM at the time of BM detection. Fractures were diagnosed in 4% (*n* = 6) and 14% (*n* = 20) needed analgesics for BM‐associated pain. In univariable analysis, patients with >5 BM experienced shorter OS from the time of BM detection compared to those with ≤5 BM (18 months vs. 75 months, *p* < .001). Among patients with Stage IV disease with and without BM, OS was shorter in patients with BM compared to patients with no BM (72 months vs. 288 months, *p* = .002). In multivariable analysis of patients with BM, higher Ki‐67% (hazard ratio [HR] = 1.06, *p* = .007), older age (HR = 1.07, *p* < .01), presence of >5 BM (HR = 1.93, *p* = .021) and synchronous BM (HR = 2.14, *p* = .016) were identified as independent prognostic factors for shorter OS. In the matched cohort of patients with Stage IV disease with and without BM, presence of BM (HR = 1.94, *p* = .009), age at diagnosis of Stage IV (HR = 1.08, *p* < .001) and locoregional surgical resection (HR = 0.47, *p* = .015) were independent prognostic factors for survival. BM are detected in approximately 25% of Si‐NET patients subjected to ^68^Ga‐DOTATOC‐PET/CT. Pain occurs in approximately 14% and fractures in 4%. The presence of BM among Stage IV patients, the extent of bone disease (>5 BM) and synchronous BM are independent prognostic factors for shorter OS.

## 1 INTRODUCTION

Current studies have reported the presence of bone metastases (BM) in about 20% of patients with NETs of different primaries.^1^ The prevalence of BM in patients with small intestinal neuroendocrine tumours (Si‐NET) ranges from 6% to 32%.^2^, ^3^, ^4^ Identification of BM has been reported to occur occasionally prior to Si‐NET diagnosis and up to 20 years after initial tumour diagnosis. However, it is usually a late event.^5^ Poorer survival outcomes have been reported for NET patients diagnosed with synchronous BM as compared to those with metachronous BM.^6^

The prevalence of BM in patients with Si‐NET is steadily increasing due to the introduction of new sensitive diagnostic imaging techniques, such as Gallium‐68‐labelled DOTA‐somatostatin analogues (^68^Ga‐SSA) as ligands for positron emission tomography (PET) and concomitant diagnostic computed tomography (^68^Ga‐DOTATOC‐PET/CT). Prior to the widespread use of ^68^Ga‐DOTATOC‐PET/CT, BM were mainly diagnosed on CT or ^111^In/^99m^Tc‐SSA scintigraphy.

The new more sensitive imaging modalities might reveal asymptomatic BM. Earlier studies have shown that at the time of BM detection, 60% of the patients reported BM‐related symptoms,^7^ mainly pain in up to 90% of patients.^2^, ^8^ However, in these studies, BM were identified on conventional radiology, showing a 9% prevalence of BM, and the presence of BM was related to more severe disease. In these earlier studies, pathological fractures because of BM are reported in less than 10% of NET patients.^2^ In general, clinical symptoms from BM, which have been diagnosed on ^68^Ga‐DOTATOC‐PET/CT, are sparsely described, especially for cohorts comprising exclusively of Si‐NET patients.

BM occur predominately in NET patients with liver metastases and are often multiple.^2^ The numbers of BM in NET patients and their prognostic impact have sparsely been studied. Sabet et al. concluded that >10 BM diagnosed on conventional CT, magnetic resonance imaging (MRI), ^111^In(^99m^Tc)‐SSA‐scintigraphy, ^68^Ga‐DOTATOC‐PET/CT or bone scintigraphy, responding well with regression on peptide radionuclide receptor treatment (PRRT), did not affect overall survival (OS),^9^ whilst a study by Deleval et al. showed shorter OS in Si‐NET patients with >5 BM diagnosed on ^18^F‐DOPA‐PET/CT.^10^ Morphological analysis has revealed that most BM from Si‐NET show both osteolytic and osteoblastic elements.^11^

Metastatic disease confers shorter OS in NETs.^12^ Liver metastases as well as BM in patients with NETs are correlated with shorter survival,^3^, ^13^ although the presence of BM has been presented with contradicting findings.^1^ A median OS of up to 60 months has been reported in NET patients with BM.^13^ Some studies suggest that patients with BM experience shorter survival,^10^, ^13^, ^14^, ^15^ while others do not with tumour‐targeted therapy.^9^ Less than 50% of NET patients with both BM and liver metastases survive >5 years, with tumour progression being the major cause of death.^16^ Importantly, most reports on BM include patients with NET of different primaries, and only a few study cohorts include solely Si‐NET patients.^10^

In this study, we hypothesize that BM in patients with Si‐NET diagnosed on ^68^Ga‐DOTATOC‐PET/CT significantly influence both clinical symptoms and survival outcomes.

## 2 PATIENT AND METHODS

### 2.1 Study population

We screened 753 Si‐NET patients, who underwent ^68^Ga‐DOTATOC‐PET/CT between 2010 and 2023 at two tertiary referral hospitals in Sweden: Uppsala University Hospital and Örebro University Hospital. Among these, 175 patients with BM were identified. Complete medical data in the patients' digital records were available for 138 patients, included in the present study. The following clinical, histopathological and imaging data were extracted for each patient: age, sex, performance status (Charlson Comorbidity Index), date of Si‐NET and BM diagnosis, primary tumour surgery, presence of liver metastases, extent of liver metastases, presence of peritoneal carcinomatosis (PC) and carcinoid syndrome (CS), presence of carcinoid heart disease (CHD) and number of BM.

WHO tumour grade and Ki‐67 index at diagnosis was noted. In the event of a subsequent tumour biopsy/biopsies, WHO tumour grade and Ki‐67 index obtained closest to the time of BM diagnosis was recorded. The liver tumour burden was classified according to an arbitrary scale: Stage I (fewer than 5 metastases in the same lobe), Stage II (bilobar and/or 5 to 10 metastases) and Stage III (more than 10 metastases or diffuse metastatic disease).

Symptoms such as pain and BM‐related fractures were registered, as well as administered analgesics and external beam radiation therapy. The number and location of BM were documented, and the patients were first divided into groups with 1, 2, 3–5, 6–10 and >10 BM. After investigating a threshold of increased mortality risk, patients with BM were further divided into two groups (≤5 BM vs. >5 BM), which was also used according to a relatively even distribution of the number of patients within the two groups.

To further evaluate the prognostic impact of BM on OS, a control group of age‐ and sex‐matched Si‐NET patients with liver metastases (Stage IV disease), but without BM, was used. The control patients were identified from the initial cohort of 753 patients who underwent ^68^Ga‐DOTATOC‐PET/CT, and a total of 77 pairs of patients were included (*n* = 154 patients with Stage IV Si‐NET, 77 patients with BM, 77 patients without BM). The baseline for survival estimate in this analysis was set from the date of Stage IV disease diagnosis.

The ^68^Ga‐DOTATOC‐PET/CT examinations were initially scrutinized by author MW. In uncertainties of radiology findings, author AS evaluated the images as well.

#### 2.1.1 ^68^Ga‐DOTATOC production

^68^Ga was obtained from a pharmaceutical grade 68Ge/68Ga generator (GalliaPharm®, Eckert & Ziegler) eluted with 0.1 M hydrochloric acid solution. 68Ga‐DOTATOC was produced using an automated synthesis platform (Modular PharmLab, Eckert & Ziegler, Germany) equipped with a disposable cassette system (C4‐Ga68‐PP) and ^68^Ga‐DOTATOC was eluted with 1 mL of 50% EtOH and formulated in sterile sodium chloride (0.9%). The subsequent sterile filtration was conducted in‐line. Radiopharmaceutical release specifications (radiochemical purity, chemical purity, quantity, solution colour, clarity) were controlled, and purity and peptide content were analysed by using high‐performance liquid chromatography with UV‐ and radio‐detectors connected in series.

#### 2.1.2 ^68^Ga‐DOTATOC‐PET/CT examination

The patients underwent PET/CT on a digital time‐of‐flight PET/CT scanner (Discovery MI, GE Healthcare, Milwaukee Wisconsin, USA in Uppsala and Siemens Biograph Vision 600, Erlangen, Germany in Örebro) utilizing clinical routine examination protocols. Whole‐body examination was performed from the proximal thighs to the base of the skull, 3 min per bed position, 1 h after i.v. injection of 2 MBq/kg body weight of ^68^Ga‐DOTATOC.

Concomitant CT was performed according to a clinical standard examination protocol whereby the liver was examined during i.v. contrast‐enhancement (Jomeprol 400 mgI/mL, 0.6 g of iodine/kg body weight, 5 mL/s) in the late arterial phase (portal‐venous inflow phase) and the neck–thorax–abdomen and pelvis were subsequently scanned in the venous contrast‐enhancement phase.

All appropriate corrections were applied to the PET data, and the PET images were reconstructed using time‐of‐flight Ordered Subset Expectation Maximization (OSEM) with 3 iterations and 16 subsets, including resolution recovery, a 5‐mm Gaussian post‐processing filter, and a matrix size of 256 × 256 with a reconstructed field‐of‐view of 50 cm.

### 2.2 Ethical considerations

This retrospective study was conducted in accordance with the Declaration of Helsinki and approved by the ethics review boards at the participating centres in Sweden (Diary number 2020‐00539) with amendments.

### 2.3 Statistical analysis

Data were registered in Microsoft Excel 2016. Nominal data are presented as number of patients and percentages, and scaled data are presented as means and medians with ranges. Kaplan–Meier survival analysis was performed to assess OS and BM stratified into ≤5 BM or >5 BM, synchronous BM and Stage IV disease, according to the first available ^68^Ga‐DOTATOC‐PET/CT with evident BM or Stage IV disease and utilized in log‐rank analysis.

Multivariate Cox regression analysis was used to determine the prognostic impact of the presence and extent of BM. A *p*‐value of <.05 was considered significant. All statistical analyses were carried out in SPSS v23.0 software package (IBM SPSS Statistics, Armonk, NY, USA).

## 3 RESULTS

**FIGURE 1:**
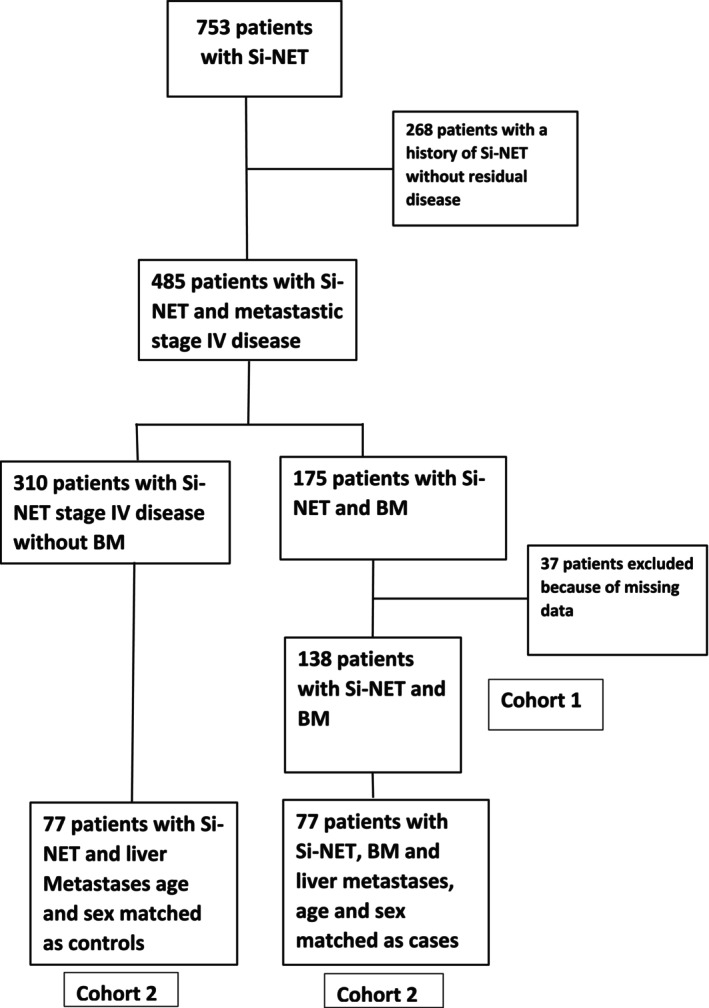
Study flow chart.

One hundred seventy‐five out of 753 patients who underwent ^68^Ga‐DOTATOC‐PET/CT (23%) were diagnosed with BM. Complete medical data were available in 138/175 patients who were included in the study. Of all the patients in the study, 36 patients presented 1 BM, 19 patients 2 BM, 22 patients 3–5 BM, 16 patients 6–10 BM and 45 patients harboured >10 BM, presented in Table 1. The patients were further subdivided into two groups: 77 patients harboured ≤5 BM and 61 patients >5 BM. The study flow chart is shown in Figure 1. The most common metastatic location was the axial skeleton, where 83% of the metastases were located, and most common in the vertebral column (27%), followed by the pelvis (19%) and the ribs (18%). No patient in this cohort presented solely distal metastases to the humerus, femur or the skull.

**TABLE 1:** Subgrouping of patients with BM.

Numbers of BM	Number of patients (*n* = 138)	Final subgroups
1	36	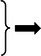 ≤5 BM (*n* = 77)
2	19	
3–5	22	
6–10	16	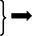 >5 BM (*n* = 61)
>10	45	

Abbreviation: BM, bone metastases.

Median age at diagnosis was 62 years (range 29–83), whereas median age at BM diagnosis was 67 years (range 49–88) and 64 patients were women (45%). Synchronous BM was seen in 46 patients (33%). All tumours were well‐differentiated; 58 were WHO Grade 1 (G1), 67 were Grade 2 (G2), 1 was Grade 3 (G3), while 12 tumours were of unspecified grade. The median Ki‐67 at diagnosis was 3% (range 1%–30%). The median (±SD) follow‐up period was 41 ± 39 months. At diagnosis, the CS was present in 48 patients (35%) and PC was encountered in 48 patients (35%), whereas hepatic metastases were present in 94 patients (68%). Among 48 patients with CS, 17 patients (35%) manifested concomitant PC. CHD was present in 31 patients (22%) and was three times more common in patients with >5 BM than in those with ≤5 BM (*p* ≤ .001). Patients with >10 BM were particularly affected by CHD compared to the other patients with less BM; thus, this was recorded separately according to the initial subdivision. In patients with >10 BM, 42% suffered from CHD (16/38).

Re‐biopsy was performed in 53 patients (38%) due to progressive disease, and 4 patients were subjected to two re‐biopsies. Only two patients had biopsies from their BM. Primary surgery was performed at NET diagnosis in 100 patients (73%). All patients were treated with SSA. Peptide receptor radionuclide therapy (PRRT) was administered to 85 patients (62%), more frequently to patients with >5 BM than those with ≤5 BM (70% vs. 55%, *p* = .048). Interferon was also more frequently administered to patients with >5 BM than to those with ≤5 BM (41% vs. 22%, *p* = .012). Treatment with everolimus was equally given to both groups, 18% (patients with ≤5 BM, *n* = 14) versus 16% (patients with >5 BM, *n* = 10). Two patients with spinal medullary compression were treated with denosumab. No patient was treated with bisphosphonates.

**FIGURE 2:**
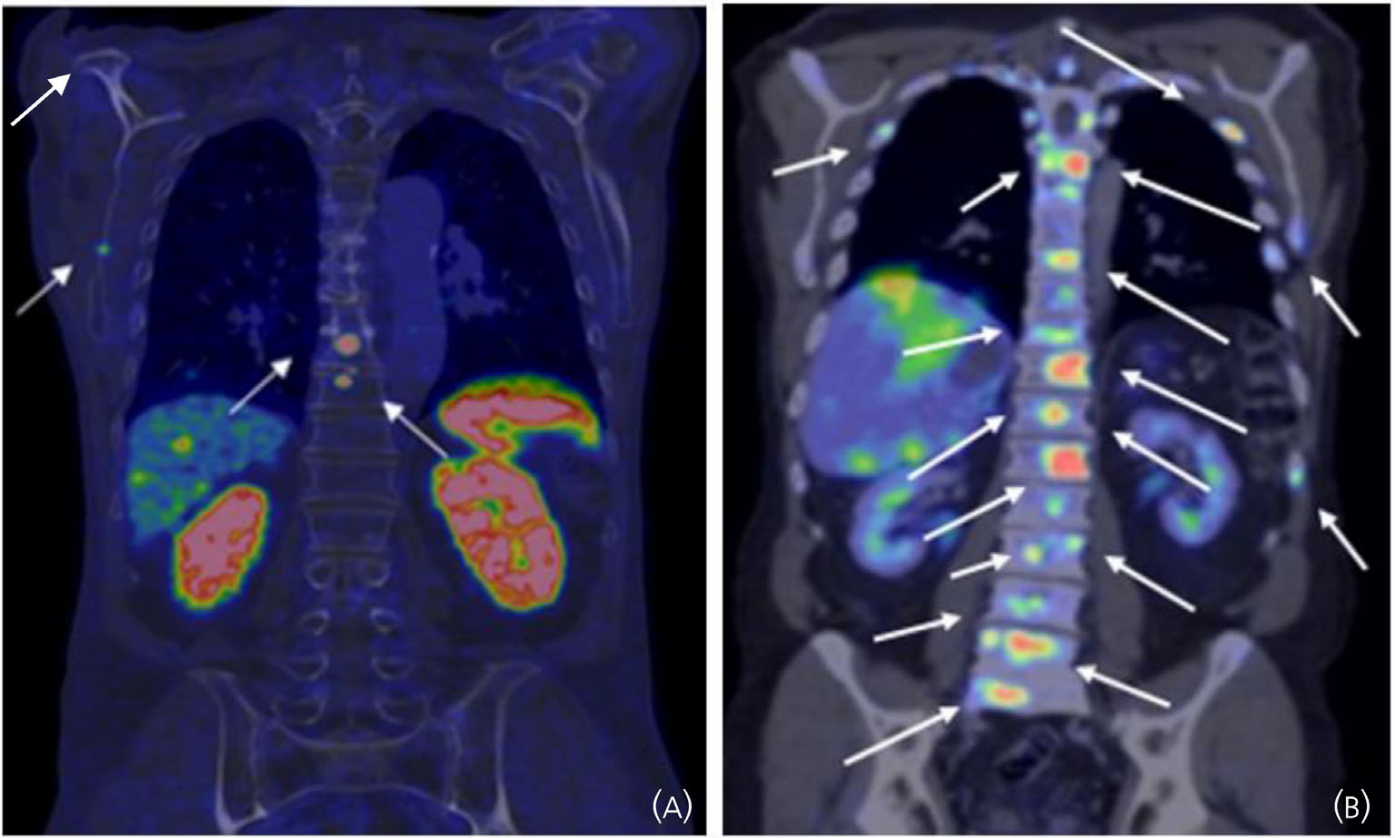
(A) ^68^Ga‐DOTATOC PET/CT (coronal PET–CT fusion) of an 83‐year‐old male with Si‐NET and ≤ 5 bone metastases indicated with arrows. (B) ^68^Ga‐DOTATOC PET/CT (coronal PET–CT fusion) of a 62‐year‐old male with Si‐NET, liver metastases and multiple bone metastases (>5) indicated with arrows. CT, computed tomography; ^68^Ga‐DOTATOC‐PET/CT, Gallium‐68‐labelled DOTA tyrosine octreotide positron emission tomography with computed tomography; PET, positron emission tomography; Si‐NET, small intestinal neuroendocrine tumour.

Baseline characteristics stratified by the extent of BM, when applying a cut‐off of five BM, are presented in Table 2. Figure 2 presents ^68^Ga‐DOTATOC‐PET/CT images of two patients with ≤5 (Figure 2A) and >5 BM (Figure 2B), respectively.

**TABLE 2:** Patient characteristics of the study cohort in patients with BM (cohort 1, *n* = 138).

*n* = 138	Total	≤5 BM	>5 BM	*p*‐value
Male gender	74 (54%)	42 (55%)	32 (53%)	.81
Female gender	64 (46%)	35 (45%)	29 (47%)	.81
Age, median in years (range)	62 (29%–83%)	61 (33%–83%)	63 (29%–83%)	.62
Ki‐67 (%), median (range)	3% (1%–30%)	3% (1%–19%)	3% (1%–30%)	.78
WHO grade				
Grade 1	58 (42%)	32 (42%)	26 (44%)	.90
Grade 2	67 (48%)	36 (47%)	31 (50%)	.98
Grade 3	1 (<1%)	0	1 (2%)	na
Missing grade	12 (9%)	9 (12%)	3 (5%)	.16
Carcinoid syndrome	48 (35%)	19 (25%)	29 (48%)	<.01
Liver metastases at NET diagnosis	94 (68%)	50 (65%)	44 (72%)	.43
None	44 (32%)	26 (34%)	18 (30%)	.59
Stage I (<5 metastases in same lobe)	20 (14%)	10 (13%)	10 (16%)	.57
Stage II (bilobar and/or 5–10 metastases)	23 (17%)	18 (23%)	5 (8%)	.02
Stage III (>10 or diffuse metastatic disease)	47 (34%)	20 (26%)	27 (44%)	.03
Missing data concerning stage	4 (3%)	2 (3%)	2 (3%)	.81
Peritoneal Carcinomatosis	48 (35%)	26 (34%)	22 (36%)	.78
BM at NET diagnosis	46 (33%)	27 (35%)	19 (31%)	.63
Carcinoid heart disease	31 (22%)	9 (12%)	22 (36%)	<.01
Years between NET diagnosis and BM	3,4 (0–37,8)	3,0 (0–37,8)	3,9 (0–19,1)	.83
Treatment				
Primary tumour surgery	100 (73%)	58 (75%)	42 (69%)	.40
SSA	138 (100%)	77 (100%)	61 (100%)	.99
PRRT	85 (62%)	42 (55%)	43 (70%)	<.01
Interferon	42 (30%)	17 (22%)	25 (41%)	<.01
Everolimus	24 (17%)	14 (18%)	10 (16%)	.78

Abbreviations: BM, bone metastases; na, not applicable; NET, neuroendocrine tumour; PRRT, peptide receptor radionuclide therapy; SSA, somatostatin analogues.

To further assess the impact of BM on OS, patients with liver metastases and BM were compared to a cohort of age‐ and sex‐matched Stage IV Si‐NET patients with liver metastases but without BM. Patient characteristics for this matched cohort are shown in Table 3.

**TABLE 3:** Patient characteristics of the two cohorts of age and sex‐matched Stage IV small intestinal neuroendocrine tumour patients with and without bone metastases (cohort 2, *n* = 154).

*n* = 154	Total	Si‐NET with BM	Controls	*p*‐value
Male gender	84 (55%)	42 (55%)	42 (55%)	.56
Female gender	70 (45%)	35 (45%)	35 (45%)	.56
Age, median in years (range)	64 (32–83)	62 (32–83)	65 (37–83)	.52
Ki‐67, median (range)	2% (1%–25%)	3% (1%–17%)	2% (1%–25%)	.84
WHO grade				
Grade 1	70 (45%)	32 (42%)	38 (49%)	.33
Grade 2	72 (47%)	37 (48%)	35 (46%)	.75
Grade 3	3 (2%)	0	3 (4%)	na
Missing grade	9 (6%)	8 (10%)	1 (1%)	.**009**
Carcinoid syndrome	61 (40%)	26 (34%)	35 (45%)	.14
Liver metastases	117 (76%)	59 (77%)	58 (74%)	.71
None	36 (24%)	17 (23%)	19 (26%)	.73
Missing data	1 (0.006%)	1 (1%)	0	na
Stage I (<5 metastases in same lobe)	21 (14%)	9 (12%)	12 (15%)	.48
Stage II (bilobar and/or 5–10 metastases)	37 (24%)	16 (21%)	21 (27%)	.35
Stage III (>10 or diffuse metastatic disease)	56 (36%)	31 (40%)	25 (32%)	.31
Missing data concerning stage	3 (2%)	3 (4%)	0	na
Peritoneal Carcinomatosis	41 (27%)	25 (32%)	16 (21%)	.10
Treatment				
Primary tumour surgery	106 (69%)	52 (63%)	54 (71%)	.73
SSA	154 (100%)	77 (100%)	77 (100%)	.99
PRRT	72 (47%)	47 (63%)	25 (38%)	.**0004**
Interferon	42 (27%)	25 (34%)	17 (21%)	.15
Everolimus	17 (11%)	5 (6%)	12 (15%)	.07
Carcinoid heart disease	26 (17%)	20 (26%)	6 (8%)	.**003**

Abbreviations: BM, bone metastases; na, not applicable; PRRT, peptide receptor radionuclide therapy; Si‐NET, small intestinal neuroendocrine tumour, SSA, somatostatin analogue.

### 3.1 Related symptoms from BM

BM‐related pain was recorded in 20 patients (14%), all of whom received analgesics, commonly paracetamol combined with opioids. Palliative external beam radiation therapy of BM was administered to 10 patients (7%). Compression of the spinal medulla was found in four patients (3%) and fractures in six patients (4%). The location of the pathological fractures was in the vertebral column and in ribs, the latter due to metastatic pleural overgrowth.

### 3.2 BM impact on OS

**FIGURE 3:**
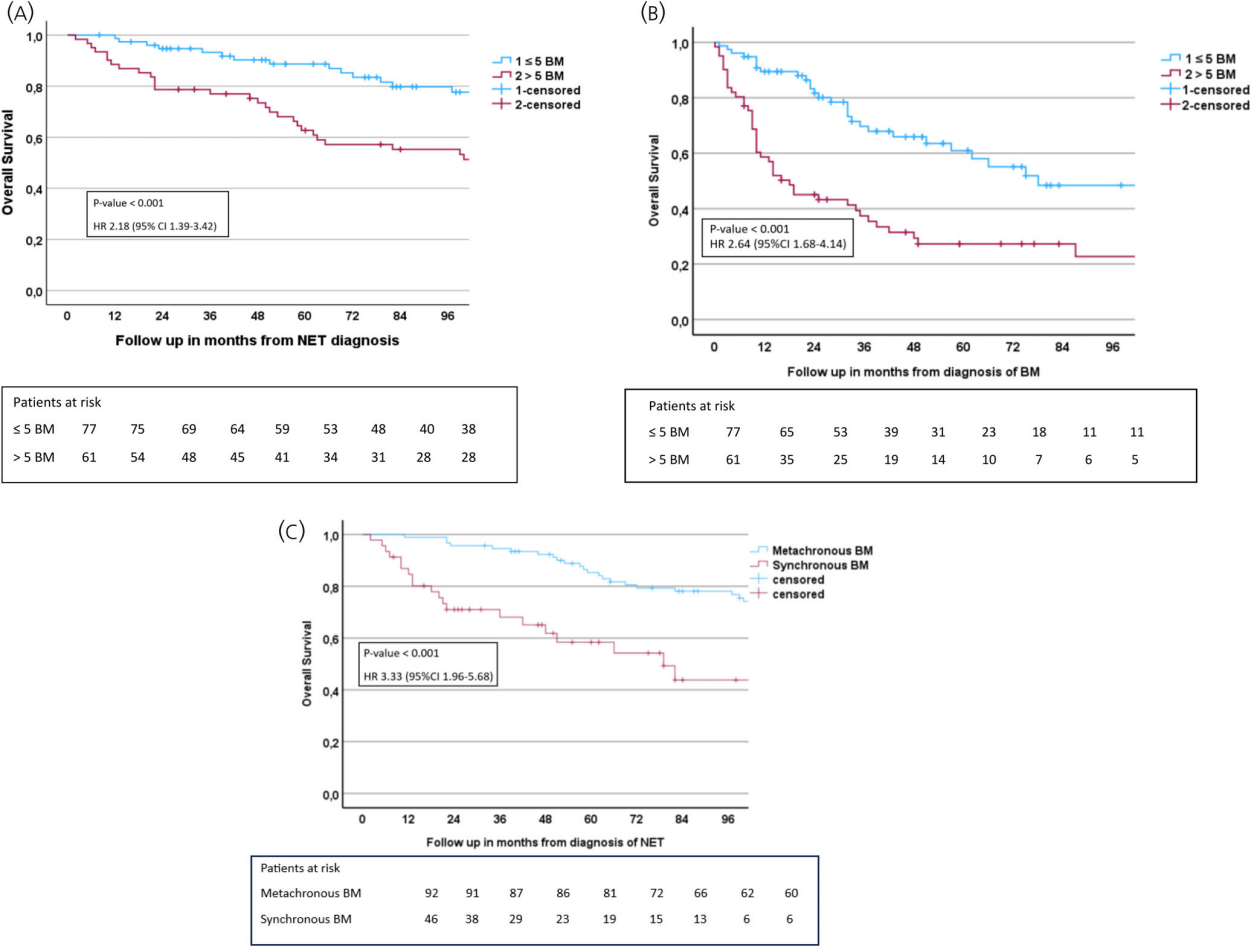
(A) Log‐rank survival analysis presenting overall survival from NET diagnosis in patients with Si‐NET and BM stratified by the number of BM (≤5 BM, blue line; >5 BM, red line). (B) Log‐rank survival analysis presenting overall survival from BM diagnosis in patients with Si‐NET and BM stratified by the number of BM (≤5 BM, blue line; >5 BM, red line). (C) Log‐rank survival analysis presenting overall survival from BM diagnosis in patients with Si‐NET and BM stratified by metachronous BM (blue line) and synchronous BM (red line). BM, bone metastases; NET, neuroendocrine tumour; Si‐NET, small intestinal neuroendocrine tumour.

Among the 138 patients with BM, the median OS was 130 months from the time of NET diagnosis and 43 months from the time of BM diagnosis. Patients with >5 BM (*n* = 61) had shorter OS than those with ≤5 BM (*n* = 77) with a median OS of 103 months versus 156 months from NET diagnosis (log‐rank *p* < .001) and 18 months versus 75 months from BM diagnosis (log‐rank *p* < .001). Patients with synchronous BM had a median OS of 79 months, whereas 145 months in patients with metachronous BM (*p* < .001). Figure 3A presents survival in time in years from diagnosis, Figure 3B from diagnosis of BM and Figure 3C metachronous versus synchronous BM from diagnosis of NET disease. Among patients with ≤5 BM, 21% (16/77) had increasing numbers of BM resulting in detection of >5 BM later during the disease. Of these, 88% (*n* = 14) were dead at the end study period.

In multivariable analysis among patients with BM, higher Ki‐67 (hazard ratio [HR] = 1.06, *p* = .007), older age (HR = 1.07, *p* < .01), presence >5 BM (HR = 1.93, *p* = .021) and synchronous BM (HR = 2.14, *p* = .016) were independent prognostic factors for shorter OS. Gender, comorbidity and primary surgery at diagnosis of NET did not affect OS (Table 4). All patients died because of progressive NET disease, except three, who died from vascular events in the brain or the heart.

**TABLE 4:** Multivariate Cox regression overall survival analysis of patients with Stage IV small intestinal neuroendocrine tumour and bone metastases (*n* = 138) from the time of diagnosis of bone metastases.

Parameter	HR (95% CI)	*p*
>5 BM	1.93 (1.11–3.35)	.021
Synchronous BM	2.14 (1.51–4.00)	.016
Age	1.07 (1.04–1.11)	<.01
Gender	0.62 (0.36–1.05)	.08
Ki‐67%	1.06 (1.02–1.11)	.007
Charlson comorbidity at diagnosis of BM	0.99 (0.83–1.17)	.86
Carcinoid syndrome	1.32 (0.76–2.31)	.34
Carcinoid heart disease	1.11 (0.62–2.01)	.73
Liver metastases	1.80 (1.00–3.24)	.05
Primary surgery	0.69 (0.34–1.40)	.30

**FIGURE 4:**
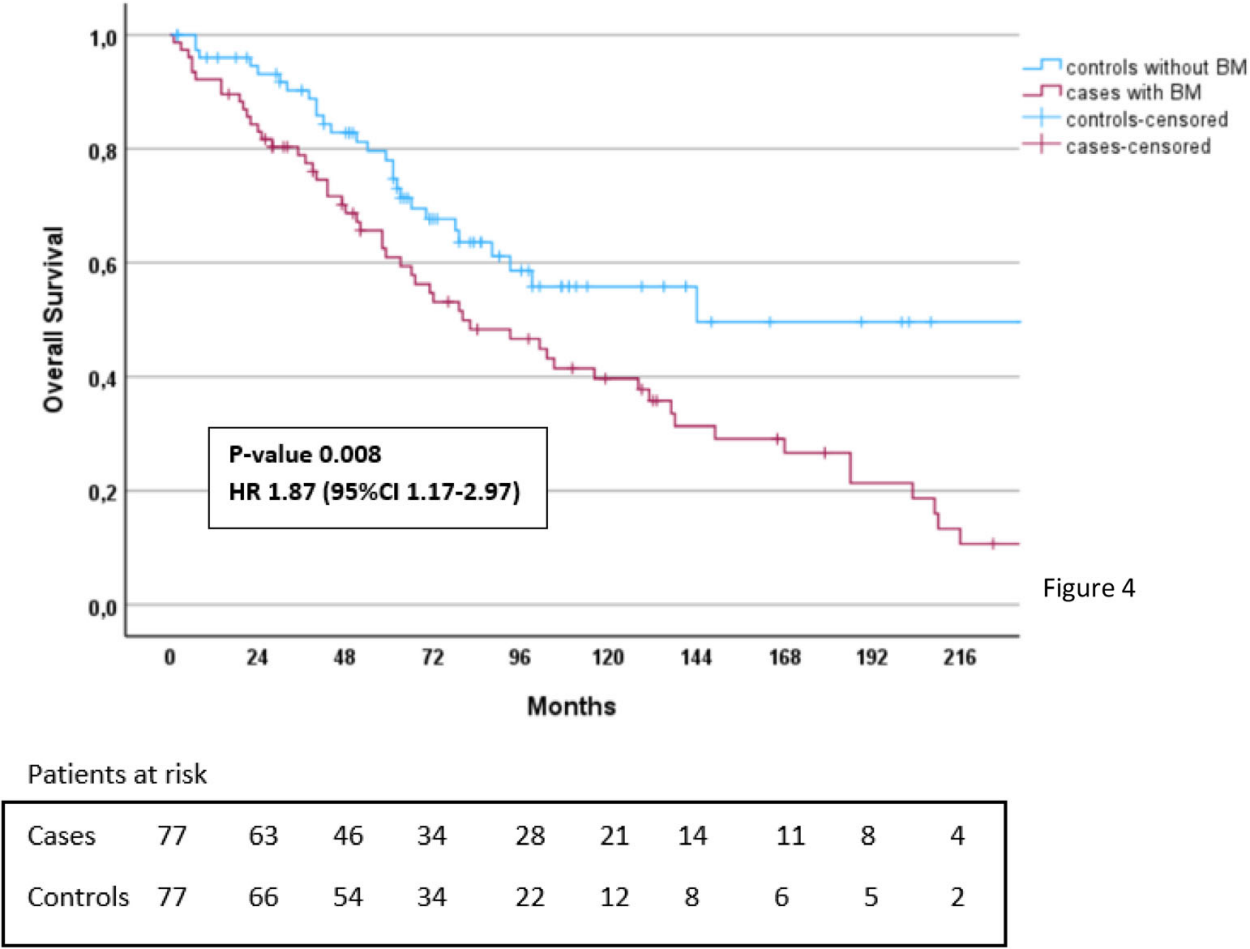
Log‐rank survival analysis of Si‐NET patients with BM at NET diagnosis (*n* = 77) and equal number of age and sex‐matched Stage IV Si‐NET patients with liver metastases only and no BM (*n* = 77). BM, bone metastases; NET, neuroendocrine tumour; Si‐NET, small intestinal neuroendocrine tumour.

Patients with BM (*n* = 77) had shorter OS compared to those with liver metastases and no BM (*n* = 77) (72 months vs. 288 months, *p* = .008), estimated from Stage IV diagnosis, presented in Figure 4.

In multivariable analysis of the matched cohorts with and without BM, age at diagnosis (HR = 1.08, *p* < .001), presence of BM (HR = 1.94, *p* = .009) and locoregional surgical resection at diagnosis (HR = 0.47, *p* = .015) were independent prognostic factors for OS (Table 5). All patients died due to progressive NET disease, except five who died from vascular events in the brain or the heart.

**TABLE 5:** Multivariate Cox regression overall survival analysis from the time of diagnosis of Stage IV disease in patients with Stage IV small intestinal neuroendocrine tumour with and without bone metastases (*n* = 154).

Parameter	HR (95% CI)	*p*
Presence of BM	1.94 (1.18–3.20)	.009
Age at diagnosis	1.08 (1.05–1.11)	<.001
Gender	0.67 (0.41–1.08)	.10
Ki‐67%	1.05 (1.00–1.10)	.07
Charlson comorbidity	1.01 (0.92–1.10)	.91
Livermetastases	1.24 (0.64–2.37)	.53
Carcinoid syndrome	1.62 (0.97–2.69)	.07
Carcinoid heart syndrome	1.51 (0.80–2.87)	.20
Primary resective surgery	0.47 (0.25–0.86)	.015

Abbreviations: BM, bone metastases; CI, confidence interval; HR, hazard ratio; NET, neuroendocrine tumour.

## 4 DISCUSSION

In our comprehensive cohort of Si‐NET patients, as many as approximately a quarter were diagnosed with BM on ^68^Ga‐DOTATOC‐PET/CT. This is a higher frequency than in previous reports, probably due to the higher sensitivity of nuclear medicine imaging, following the introduction of ^68^Ga‐labelled DOTA‐SSA as ligands for PET/CT.

BM‐related pain occurred in 14% of our patients, who all received analgesics, whereas 7% were treated with palliative external beam radiation. Compression of the spinal medulla was seen in 3% and fractures in 4%. Earlier studies on the symptomatology of NET‐related BM are sparse. In a previous study, only 9% of patients had BM; however, this study was limited by the fact that BM were detected by conventional radiology,^17^ with inferior sensitivity as compared to ^68^Ga‐DOTATOC‐PET/CT.^1^, ^5^ Symptomatic BM, detected on CT scan, were earlier described in up to 42% of patients suffering from pain and fractures.^17^ By contrast, all BM were diagnosed on ^68^Ga‐DOTATOC‐PET/CT in our study, and symptoms were much less frequent. A recent review regarding the detection of BM on ^68^Ga‐DOTATOC‐PET/CT reported approximately 90% sensitivity and almost maximum specificity.^18^ False‐positive results with ^68^Ga‐DOTATOC‐PET/CT may occur in patients with inflammatory processes, meningiomas, lymphomas, angiomas or tumours other than NETs expressing somatostatin receptors.^13^ Interestingly, NET‐related BM appear to involve the axial rather than the appendicular skeleton.^17^, ^19^

Regarding the prognostic impact, we found that patients with BM had a median OS of 130 months from NET diagnosis and 43 months from BM diagnosis, implying that BM could be a late event in the clinical course of the disease. OS was also significantly decreased in patients with >5 BM compared to those with ≤5 BM (18 months vs. 75 months, *p* < .001) from time at skeleton diagnosis, indicating the prognostic value of the number of BM. Synchronous BM also impaired survival compared to metachronous BM (79 months vs. 145 months, *p* < .001). In addition, in Stage IV disease, the median OS was shorter for patients with BM compared to those without BM (72 months vs. 288 months, *p* = .002). Similar results have been shown in the recent literature, reporting shorter OS in patients with BM, with a median OS of 49 months, as compared to 101 months in those with other locations of NET metastases.^17^

The prognostic role of BM as an independent negative predictor for survival was confirmed in a multivariable analysis along with the extent of bone disease. Though, BM do not seem to cause a high number of fractures and associated hospitalization complications in our patient cohort, accounting for direct mortality.

The impact on survival of locoregional surgical resection of the primary tumour in Stage IV disease is controversial with contradicting results in previous studies.^1^, ^3^, ^13^ Thus, our results on surgical management of the primary Si‐NET in patients with BM should be interpreted with caution. In the multivariable analysis of patients with Stage IV disease, we found a positive effect on survival in patients who underwent surgery at the time of Stage IV diagnosis irrespective of the presence of BM. Another study reported a significantly longer OS for patients with BM undergoing surgical resection of the primary tumours than those who did not (median OS 76 months vs. 32 months, *p* = .005).^19^ However, in these analyses, there was an inherent selection and immortal bias since the surgical management of the primary tumour was not chosen as the primary endpoint, and neither were the survival estimates assessed from the time of surgery.

Interestingly, CHD was present in 22% of our patients with BM, especially patients with >10 BM presenting a prevalence of CHD up to 42%. Generally, an approximately 6% prevalence of CHD has been described in previous literature, which is in line with our study in the subset of Stage IV patients without BM.^20^ Although there is an association between CHD and liver metastases, our study implies that extensive extrahepatic metastatic disease, in particular BM, could be associated with CHD.

The therapeutic approach to BM in Si‐NET patients is not well established. Treatment in disseminated NET disease is palliative, with the aims to prolong progression‐free survival and to reduce the NET symptoms and pain. Effective treatment of disseminated disease indeed requires a multimodal approach. SSA constitute an established symptomatic and antiproliferative treatment in well‐differentiated NET.^21^, ^22^ The efficacy has been confirmed in clinical trials such as PROMID^22^ and CLARINET.^21^ Treatment with (INF‐α) has shown similar effects as SSA.^23^ Due to adverse effects such as fatigue, weight loss, fever and so forth, INF‐α is currently less frequently used.^23^ PRRT has in recent studies been shown to inhibit tumour progression and increase OS in patients with metastatic NET.^24^ To date, PRRT is considered a commonly used therapy for patients with well‐differentiated, metastatic, progressive, somatostatin receptor positive GEP‐NETs.^24^ Studies have shown that PRRT induced an objective response in bone lesions in up to 50% of NET patients with BM.^9^

Bone‐directed therapies include orthopaedic surgery, external beam radiation therapy, bisphosphonates and denosumab. Bone pain relief has been described in up to 90% of patients treated with external beam radiation therapy.^25^ Both orthopaedic surgery and external beam radiation therapy are indicated to prevent and treat severe bone‐related events, such as spinal cord compression.^26^ However, in our comprehensive cohort of 138 patients, only four experienced medulla compression, and fractures were seen in six. Twenty patients (14%) were recorded with BM‐related pain, which responded well to analgesics (paracetamol and opioids). External beam radiation therapy towards BM was given in 10 patients for palliation. Thus, despite the common event of BM in our unselected group of Si‐NET, only a few patients experienced symptoms that needed clinical intervention. Of special interest is that none of them received treatment with bisphosphonates.

Our study has some limitations, including its retrospective nature and multi‐centre design. Thus, different surveillance approaches, with variable time intervals for functional imaging in the two participating centres, may confound the results regarding the time of BM detection and setting the baseline for survival estimates. There is also a risk of referral bias in our study, since tertiary referral centres may have included patients with more advanced disease. Furthermore, in this type of study it was, because of ethical and logistical reasons, not possible to biopsy and achieve histopathological verification of all BM diagnosed on ^68^Ga‐DOTATOC‐PET/CT. Therefore, there is a theoretical risk of false positive detections of BM, owing to ^68^Ga‐DOTATOC uptake in inflammatory lesions or in tumours other than NET.

A strength of our study was the homogeneous and large cohort of Si‐NET patients with BM, and that the detection of these metastases was based solely on ^68^Ga‐DOTATOC‐PET/CT, currently the most sensitive and specific imaging technique for diagnosing BM in Si‐NET patients. By contrast, previous studies with various NET primaries have presented BM on different imaging modalities, which hampers comparisons between these reports and with the more recent studies. Hence, this might further explain our higher prevalence but lower rate of symptomatic BM than in earlier studies.

Also, we excluded patients with poorly differentiated NECs and NETs of other primary origin than the small intestine to minimize tumour heterogeneity in our cohort. Although central nuclear medicine imaging review was not undertaken in all patients, expert reading of ^68^Ga‐DOTATOC‐PET/CT by author AS was performed in cases of any uncertainty. We do not have sufficient FDG‐PET CT data in our cohort to make pertinent analyses and clinical inferences since Si‐NETs are generally low‐proliferative tumours.

Given that the current detection rate of BM is higher than in previous reports, larger multicentre studies in patients with NETs may better delineate the clinical impact of BM and suggest more advantageous treatment strategies, particularly among emerging bone‐specific treatments.

## 5 CONCLUSIONS

^68^Ga‐DOTATOC‐PET/CT constitutes a highly sensitive modality detecting BM in as many as approximately a quarter of Si‐NET patients. Synchronous disease and >5 BM adversely affect the prognosis of Si‐NET and constitute independent negative prognostic factors for survival. Symptoms from skeletal metastases are relatively rare (pain 14%, fractures in 4%). Additionally, the presence of BM in Stage IV disease results in significantly worse prognosis than in patients without BM.

## AUTHOR CONTRIBUTIONS

**Maria Wedin:** Data curation; formal analysis; investigation; methodology; project administration; software; resources; validation; visualization; writing – review and editing; writing – original draft. **Eva Tiensuu Janson:** Methodology; formal analysis; supervision; visualization; writing – original draft; writing – review and editing. **Göran Wallin:** Formal analysis; project administration; methodology; writing – original draft; writing – review and editing. **Anders Sundin:** Supervision; data curation; formal analysis; project administration; visualization; validation; investigation; writing – original draft; writing – review and editing. **Kosmas Daskalakis:** Methodology; validation; visualization; writing – review and editing; writing – original draft; formal analysis; supervision; project administration.

## CONFLICT OF INTEREST STATEMENT

The authors declare no conflicts of interest.

## PEER REVIEW

The peer review history for this article is available at https://www.webofscience.com/api/gateway/wos/peer-review/10.1111/jne.70073.

## Data Availability

The data that support the findings of this study are available from the corresponding author upon reasonable request.
